# Effects of quercetin-conjugated with superparamagnetic iron oxide nanoparticles on learning and memory improvement through targeting microRNAs/NF-κB pathway

**DOI:** 10.1038/s41598-020-71678-4

**Published:** 2020-09-15

**Authors:** Shiva Ebrahimpour, Abolghasem Esmaeili, Fariba Dehghanian, Siamak Beheshti

**Affiliations:** 1 Department of Cell and Molecular Biology and Microbiology, Faculty of Biological Science and Technology, University of Isfahan, HezarJarib Street, 81746-73441 Isfahan, Iran; 2 Department of Plant and Animal Biology, Faculty of Biological Science and Technology, University of Isfahan, HezarJarib Street, 81746-73441 Isfahan, Iran

**Keywords:** Computational biology and bioinformatics, Molecular biology, Neuroscience, Diseases, Nanoscience and technology

## Abstract

Quercetin-conjugated superparamagnetic iron oxide nanoparticles (QCSPIONs) have an ameliorative effect on diabetes-induced memory impairment. The current study aimed to compare the effect of quercetin (QC) and QCSPIONs on inflammation-related microRNAs and NF-κB signaling pathways in the hippocampus of diabetic rats. The expression levels of miR-146a, miR-9, NF-κB, and NF-κB-related downstream genes, including TNF-α, BACE1, AβPP, Bax, and Bcl-2 were measured using quantitative real-time PCR. To determine the NF-κB activity, immunohistochemical expression of NF-κB/p65 phosphorylation was employed. Computer simulated docking analysis also performed to find the QC target proteins involved in the NF-κB pathway. Results indicate that diabetes significantly upregulated the expression levels of miR-146a, miR-9, TNF-α, NF-κB, and subsequently AβPP, BACE1, and Bax. Expression analysis shows that QCSPIONs are more effective than pure QC in reducing the expression of miR-9. Interestingly, QCSPIONs reduce the pathological activity of NF-κB and subsequently normalize BACE1, AβPP, and the ratio of Bax/Bcl-2 expression better than pure QC. Comparative docking analyses also show the stronger binding affinity of QC to IKK and BACE1 proteins compared to specific inhibitors of each protein. In conclusion, our study suggests the potent efficacy of QCSPIONs as a promising drug delivery system in memory improvement through targeting the NF-κB pathway.

## Introduction

Diabetes mellitus (DM) is a growing metabolic syndrome with long-term vascular complications including nephropathy, retinopathy, and neuropathy^1^. Diabetic patients usually present central nervous system (CNS) manifestations including brain-tissue atrophy, hippocampal size reduction, alteration in electrophysiological properties, reduction of neurogenesis, and loss of synaptic plasticity that eventually result in deficits in cognitive performance^2–5^. Several mechanisms, including beta-cell dysfunction with lower circulatory insulin levels, insulin resistance (IR), yperglycemia, oxidative stress, adipokines dysregulation, mitochondrial dysfunction, and inflammation have been involved in diabetes complications and can be classical targets to reduce diabetic complications^6, 7^. Hyperglycemia, through several mechanisms including glucose-mediated activation of protein kinase c (PKC) isoform, aldose reductase (AR) pathway, increased production of reactive oxygen species (ROS), and extra production of advanced glycation end products (AGEs) can cause nuclear translocation of NF-κB and generation of proinflammatory cytokines and further neuroinflammatory process^8, 9^. Besides, there is a mutual cross-talk between central and peripheral inflammation^10^. However, the brain is an immune-privileged system, in diabetes conditions, peripheral inflammatory cytokines such as TNF-α transfer from the blood–brain barrier (BBB) and act as the early stimuli to the brain microglia^11^. TNF-α leads to stimulation IKKβ/NF-κB pathway that causes serine kinase phosphorylation of insulin receptor substrates (IRSs), which can block insulin signaling and eventually lead to the occurrence of IR. in fact, NF-κB plays a fundamental relationship between IR and inflammation in IR-related diseases such as DM^12^. On the other hand, numerous mechanisms have been proposed to describe in what manner IR and hyperinsulinemia can cause AD. (i) the activation of the MAPK signaling pathway and enhancement of expression of beta-secretase 1 (BACE1) is stimulated by IR and hyperinsulinemia which as a final point accumulate abnormal Aβ peptides and neuritic plaques^13^. (ii) the stimulation of the Akt pathway is reduced by IR and then dephosphorylation and GSK3β activation are occurred^14^. (iii) IR sequestrate the insulin-degrading enzyme (IDE), a key enzyme in Aβ degradation, and thus decreases the Aβ clearance and facility of its aggregation^15^. (iv) IR inhibits protein phosphatase 2A (PP2A) and subsequently accumulate tau hyperphosphorylation and NFTs^16^.

MicroRNA-146a (miR-146a) is the most known inflammation-sensitive microRNA that acts as a negative regulator of inflammation^17^. Some recent studies uncovered that NF-κB activation increased miR-146a expression, in turn, prevented the translation of IRAK1 and TRAF6 and led to feedback on this pathway^18–21^. MicroRNA-9 is another brain-rich miRNAs involved in cellular functions and brain development^17, 22^. IL-1β and TNF-α mediate miR-9 that can act as a fine-tuning mechanism in inflammatory processes by targeting NF-κB^18, 23^. NF-κB has been introduced as a definitive and complex player in the immune response modulation, which is involved in the pathogenesis a wide range of disorders^24^. The dual role of NF-κB in cognition processes and inflammatory cascades made NF-κB and its upstream and downstream molecular pathways as proper targets for early intervention in the treatment of various neurological disorders^25^.

Quercetin (3,3′,4′,5,7-pentahydroxyflavone, (QC)), as a flavonoid available in the daily diet, possess potent anti-inflammatory, antioxidant, anti-cancer, anti-allergic and antiviral activities^26, 27^. Achieved evidence from cell cultures and animal models shows the anti-diabetic effects of quercetin via hypoglycemic effects, stabilization of long sustaining insulin secretion, regeneration of human islets in the pancreas, and reduction of IR^28–31^. These qualities are considered favorable for protecting cells from damages in many organs such as brain^32^. Emerging studies reported that QC can provide beneficial effects against different diseases by influencing gene expression at the transcriptional level, epigenetic level, and post-transcriptional level by modulating miRNAs^34, 35^. Despite all of the pharmacological properties, low aqueous solubility and stability, poor permeability, and slight oral bioavailability limit QC clinical application^36^. QC-loaded nanoparticles significantly enhanced the bioavailability and protective effects of QC in the brain by reducing loss in the gastrointestinal tract within oral administration^37^. In a previous report, we revealed that oral delivery of QC-conjugated superparamagnetic iron oxide nanoparticles (QCSPIONs) had a significantly better effect on the improvement of memory performance in diabetic rats as compared to pure QC^38^.

The current report focused on the miRNAs/NF-κB pathway as a molecular mechanism of the neuroprotective effect of QCSPIONs against memory dysfunction. Inflammation is a critical link between diabetes and CNS pathology and treatments that inhibit and target NF-κB always improve IR, beta-cell damage and apoptosis, and other diabetic complications^7, 39^. Given the significant role of miR-9 and miR-146a in the regulation of NF-κB-dependent neuroinflammatory pathway, they were considered as two candidate miRNAs^40^. Although several recent studies have indicated the involvement of miR-146a and miR-9 in the pathogenesis of diabetic complications^21, 41^, there is no report about the effect of QC on these microRNAs and related genes in diabetic neuropathy. Thus, the goal of the current study is to compare the effects of QC and QCSPIONs on the expression of miR-146a, miR-9, NF-κB, and the activity of NF-κB in the hippocampus of diabetic rats. Furthermore, we examined the mRNA expression levels of BACE1, ABPP, BAX, BCL2, and TNF-α as NF-κB target genes to clarify a clue to understanding the effect of QCSPIONs on NF-κB-dependent inflammatory pathways in learning and memory. Further molecular docking study was also performed to find out the potential inhibitory effects of QC on proteins of NF-κB signaling compared to different drugs targeting specifically NF-κB members.

## Results

### Synthesis and characterization of QCSPIONs

As we reported before^38, 42^ the observation of certain vibration peaks at 3,378 cm^−1^ and 573 cm^−1^, representing O–H and Fe–O bonds, respectively, in the Fourier transform infrared (FTIR) spectrum of dextran-coated Fe_3_O_4,_ confirmed the coating of SPIONs with dextran and magnetite spinel structure. The FTIR of QC showed vibration peaks at 3,388 cm^−1^, 1657 cm^−1^, and 1,150–1,070 cm^−1^ are attributed to O–H, C=O, and C–O bonds, respectively. Additionally, a peak of 933 cm^−1^ displays C-H bending vibration of aromatic groups. Given the presence of this peak and other characteristic peaks in the FTIR spectrum of the QCSPIONs the conjugation of QC to SPIONs was successfully performed.

The crystalline structure of dextran-coated Fe_3_O_4_ and QCSPIONs were studied by X-ray diffraction (XRD) analysis. The presence of diffraction peaks at 30.1°, 35.4°, 43.9°, 53.4°, 57.0°, and 62.6° verified the crystalline structure of the magnetite.

The size and the exterior morphology of QCSPIONs were visualized by field emission–scanning electron microscope (FE–SEM) data. The SEM result estimated the diameter of NPs in the range of 30–50 nm. In addition, two certain peaks of oxygen and iron in the EDX spectroscopy confirmed the SPIONs identity. All of the pictures presented in our previous studies.

### The entrance of SPIONs into tissues

**Figure 1:**
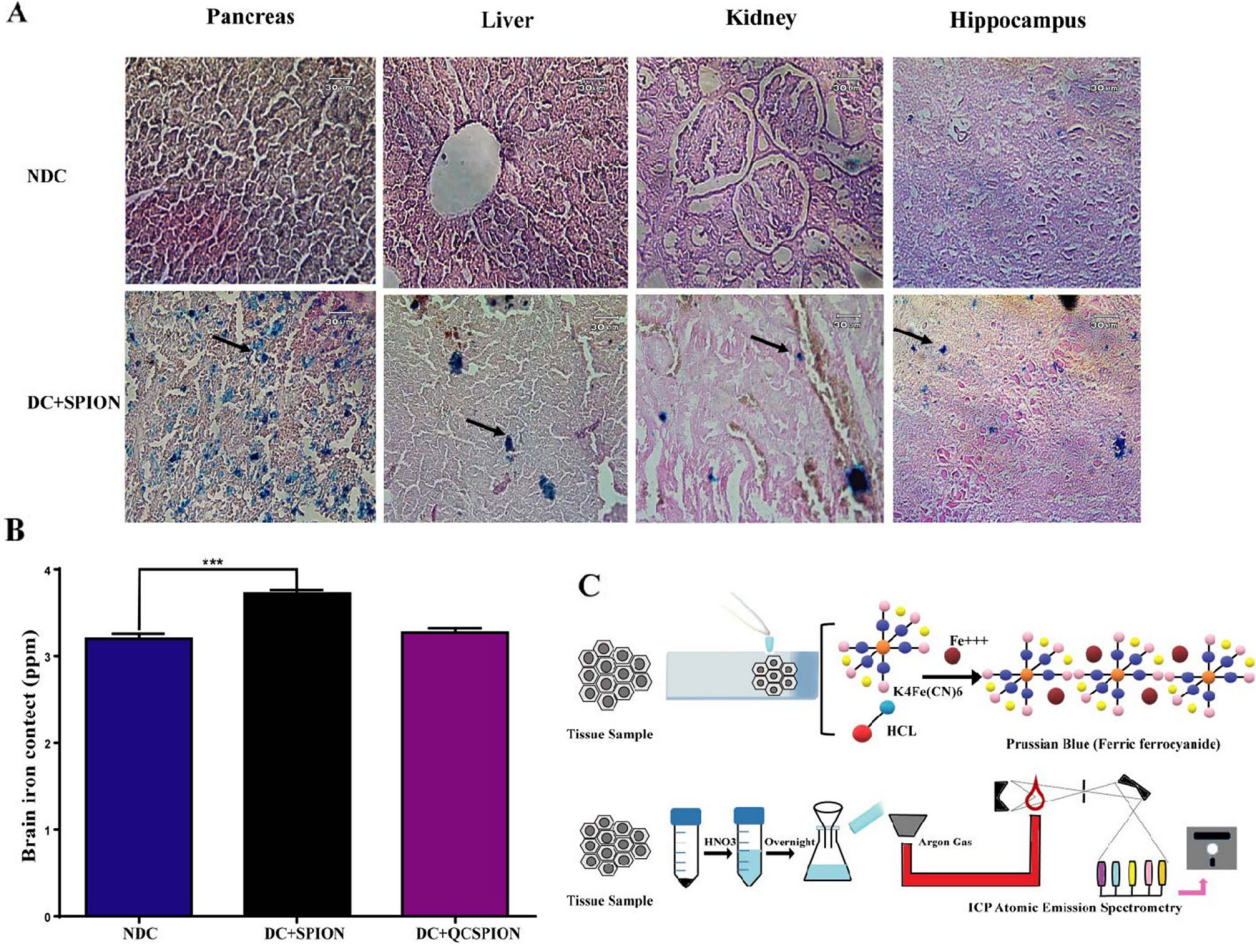
Results of Prussian blue staining and ICP-AES. (**A**) Prussian blue staining of pancreas, liver, kidney, and hippocampus tissues of NDC and DC treated with SPION. (**B**) ICP results obtained of hippocampus tissue in NDC and DC treated with SPION and QCSPION. (**C**) Schematic picture of Prussian blue staining and ICP assay. NDC: non-diabetic control, DC, diabetic control, DC + SPION: diabetic treated with superparamagnetic iron oxide nanoparticle, DC + QCSPION: diabetic treated with quercetin-conjugated superparamagnetic iron oxide nanoparticle. *** P < 0.001 versus the diabetic control group (one-way ANOVA, Tukey’s multiple comparison tests). Arrow represents the position of the iron oxide nanoparticles. (scale bar: 30 μm, magnification 40X).

The accumulation of QCSPIONs in the hippocampus of rats was investigated by Prussian blue staining (Fig. 1A). We found a large number of blue dots in the hippocampus of diabetic rats administered with SPIONs which indicating their ability to pass through the BBB and absorption by the nervous system cells in diabetic conditions. Prussian blue staining of pancreas, liver, and kidney tissues of control and SPIONs groups was also done and the gained results showed that more iron accumulations in the liver and kidney of groups treated with SPIONs as compared to the control group. Interestingly, the uptake of these SPIONs in the pancreas appeared to be higher than other tissues which may be due to the vascular permeability of pancreatic islets after administration of STZ.

To further verify uptake of nanoparticles into the brain of rats, the iron accumulation in the hippocampus 35 days after treating with SPIONs and QCSPIONs was quantified by inductive coupled plasma-atomic emission spectrometer (ICP-AES). As shown in Fig. 1B, iron content in control rats was 3.203 ± 0.05 ppm, whereas in diabetic rats treated with SPIONs and QCSPIONs were 3.723 ± 0.03, 3.270 ± 0.05 ppm, respectively. This data revealed a significant difference in the iron concentrations between the control and SPIONs treated rats (*P* = 0.0009) after five days from the last daily gavage, which is consistent with Prussian blue staining results. Although the concentration of iron in the hippocampus of QCSPIONs group was greater than the control, the alteration was not significant which may be due to a lower concentration of iron in the conjugated form.

### Quantitative real-time PCR analysis

**Figure 2:**
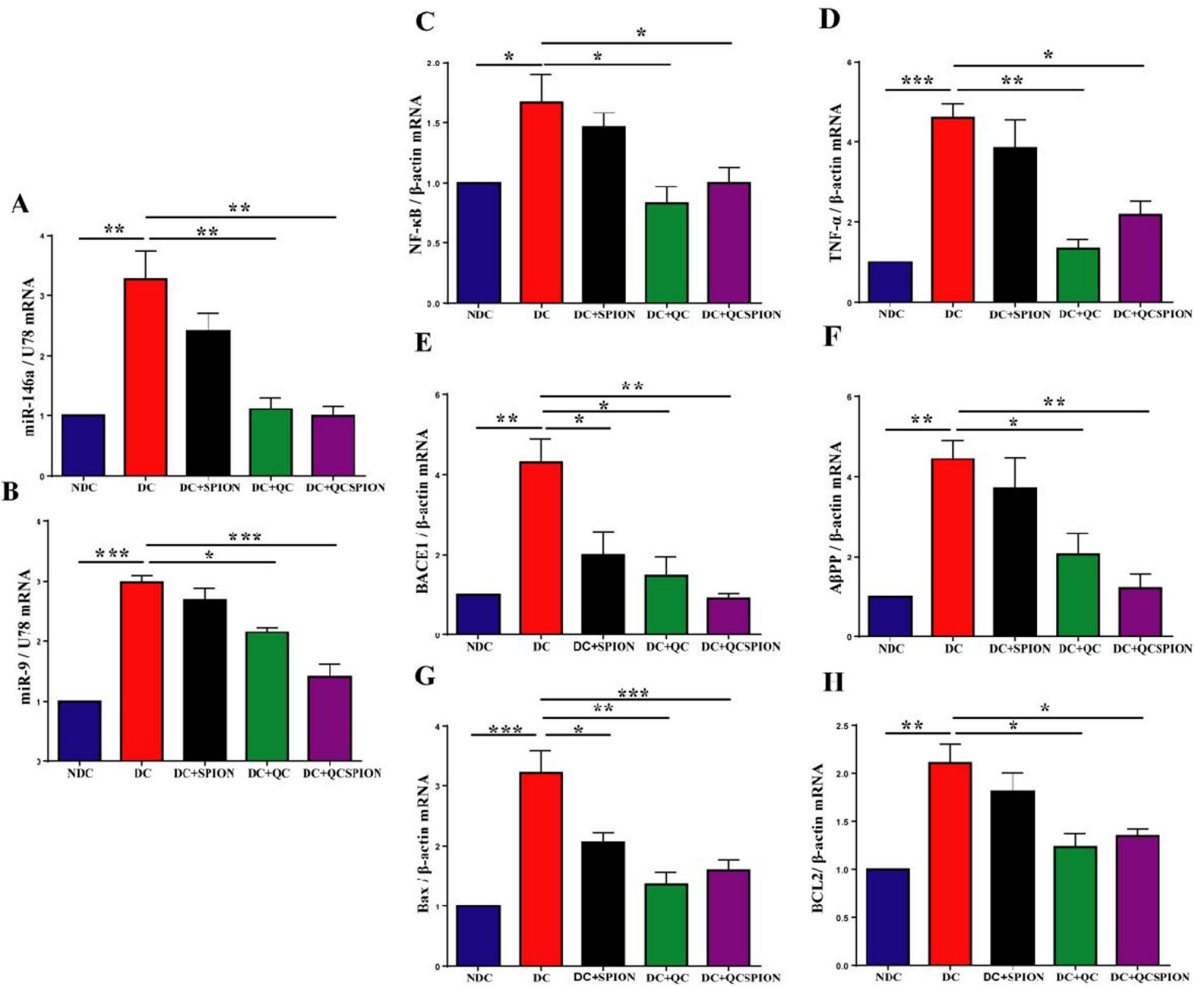
The graph of relative expression of (**A**) miR-146a, (**B**) miR-9 (**C**) NF-κB, (**D**) TNF-α, (**E**) BACE1 (**F**) AβPP (**G**) Bax, and (**H**) Bcl-2 in the hippocampus of experimental groups. NDC: non-diabetic control, DC: diabetic control, DC + SPION: diabetic treated with superparamagnetic iron oxide nanoparticle, DC + QC: diabetic treated with quercetin, DC + QCSPION: diabetic treated with quercetin-conjugated superparamagnetic iron oxide nanoparticle. * P < 0.05, ** P < 0.01 and *** P < 0.001 and *P* < 0.0001 versus diabetic control group (one-way ANOVA, Tukey’s multiple comparison tests). The expression levels are investigated by quantitative real-time PCR and ΔΔCt method. Data expressed as mean ± S.E.M.

The genes expression level of miR-146a, miR-9, NF-κB, TNF-α, BACE1, AβPP, Bax, and Bcl-2 in the hippocampus of rats are shown in Fig. 2A–H. Expression analysis indicated the expression levels of miR-146a and miR-9 in the hippocampus of the diabetic rats were approximately threefold more than control rats fifty days after induction of diabetes (fold change = 3.27, *P* = 0.0010 for miR-146a and fold change = 2.98, *P* < 0.0001 for miR-9). Tukey's multiple comparisons test showed a significant decrease in the expression level of miR146a by QC in pure (fold change = 1.10, *P* = 0.0015) and conjugated forms (fold change = 0.1, *P* = 0.0010). Similarly, QC application decreased the miR-9 expression level (fold change = 2.14, *P* = 0.0108); however, the most significant effect was observed in the QCSPIONs-treated group (fold change = 1.41, *P* < 0.0001). In addition, real-time PCR profiles indicated an increase in the mRNA expression levels of NF-κB (fold change = 1.66, *P* = 0.0492) and TNF-α (fold change = 4.59, *P* = 0.0006) in the hippocampus of diabetic rats. Administration of QC and QCSPIONs significantly decreased expression levels of NF-κB (fold change = 0.83, *P* = 0.0135 for QC and fold change = 1.000, *P* = 0.0495 for QCSPIONs) and TNF-α (fold change = 1.33, *P* = 0.0013 for QC and fold change = 2.1, *P* = 0.0108 for QCSPIONs) in the diabetic group in comparison with corresponding controls. These results showed that QC and QCSPIONs had the same effect on the reduction of NF-κB gene expression but, QC was more effective than QCSPIONs in reducing the level of TNF-α. Interestingly, One-way ANOVA results revealed that expression levels of BACE1 and AβPP were increased approximately fourfold in diabetic rats (fold change = 4.29, *P* = 0.0021 for BACE1 and fold change = 4.43, *P* = 0.0044 for AβPP). QCSPIONs restored the excessive expression levels of BACE1 and AβPP towards control values better than pure QC (fold change = 0.91, *P* = 0.0017 for BACE1 and fold change = 1.20, *P* = 0.0067 for AβPP). Surprisingly, Fe_3_O_4_ nanoparticles significantly reduced mRNA expression levels of BACE1 in the STZ group (fold change = 2, *P* = 0.0233), suggesting the improvement effect of Fe_3_O_4_ NPs on memory. Further analysis demonstrated hyperglycemia caused a significant enhancement in the expression levels of Bax (fold change = 3.22, *P* = 0.0002) and Bcl-2 (fold change = 2.10, *P* = 0.0021) in the diabetic group, whereas in the case of QC and QCSPIONs treatments, the expression of Bax (fold change = 1.35, *P* = 0.0009 for QC and fold change = 1.59, *P* = 0.0025 for QCSPIONs) and Bcl-2 (fold change = 1.23, *P* = 0.0086 for QC and fold change = 1.34, *P* = 0.0150 for QCSPIONs) were significantly downregulated, which are almost the same level as the control group. Overall, the results obtained from quantitative real-time PCR analysis indicated that diabetes significantly changed the expression levels of all genes studied, but the application of QC in pure and especially conjugated forms normalized the expression of the genes in the hippocampus of diabetic rats.

### Immunohistochemical analysis

**Figure 3:**
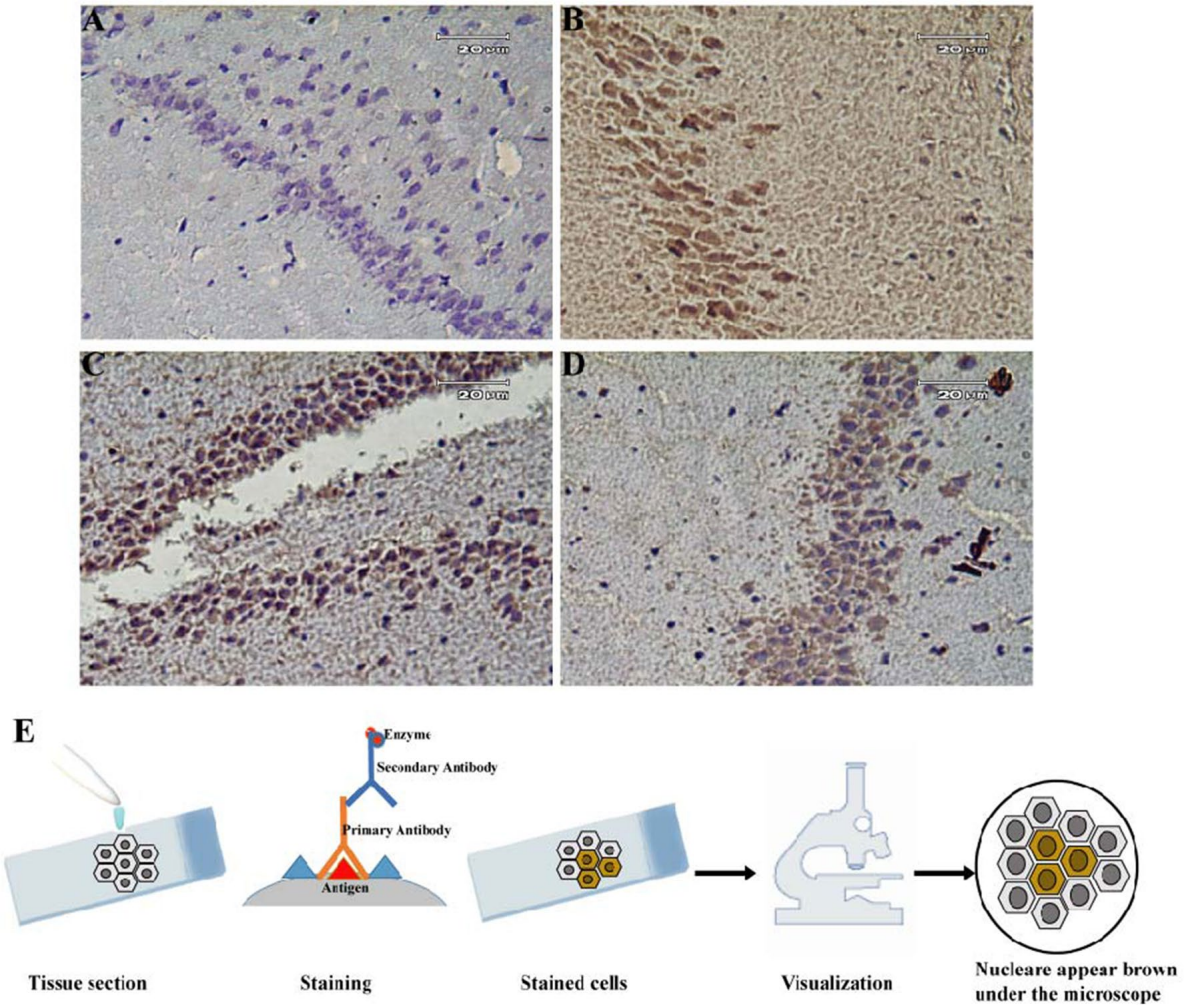
Representative photomicrographs of immunohistochemistry staining with antiphospho-NF-κB p65 antibody in the hippocampus of different groups. (**A**) NDC rats showing no phospho-p65 positive cells, (**B**) DC rats showing a significant increase in a number of phospho-p65 positive cells, (**C**) DC + QC demonstrating a reduction in NF-κB immunoreactivity, (**D**) DC + QCSPION showing a significant reduction in activated NF-κB signal. (**E**) Schematic picture of IHC. NDC: non-diabetic control, DC: diabetic control, DC + QC: diabetic treated with quercetin, DC + QCSPION: diabetic treated with quercetin-conjugated superparamagnetic iron oxide nanoparticle. (scale bar: 20 μm, magnification 40X). Brown color indicates NF-κB positivity.

The effect of miR-146a on the NF-κB activity in the hippocampus of male Wistar rats, immunohistochemical staining by an antibody versus activated NF-κB, anti phospho- NF-κB p65 (S536), was performed. In the normal brain tissue, only a few phospho-p65 positive cells were identified, whereas the number of phospho-p65 positive cell considerably elevated in the hippocampus nucleus of diabetic rats, verifying the activation of NF-κB after treating with STZ (Fig. 3A,B). The numbers of the phospho-p65 positive cell significantly reduced in both QC and QCSPIONs treated groups as compared to the diabetic group; however, the most favorable effect was created by QCSPIONs treatment (Fig. 3C,D). As shown in Fig. 3, the p65 (phospho S536) signal was mostly observed in the nucleus of the cells and more noticeably in the CA3 region of the hippocampus and amygdaloid nuclear complex, suggesting the key role of the hippocampus in brain inflammation and diabetic-related cognitive impairment.

### Docking calculations indicate the significant inhibitory effects of QC on the NF-κB pathway through targeting IKK and BACE1 proteins

**Figure 4:**
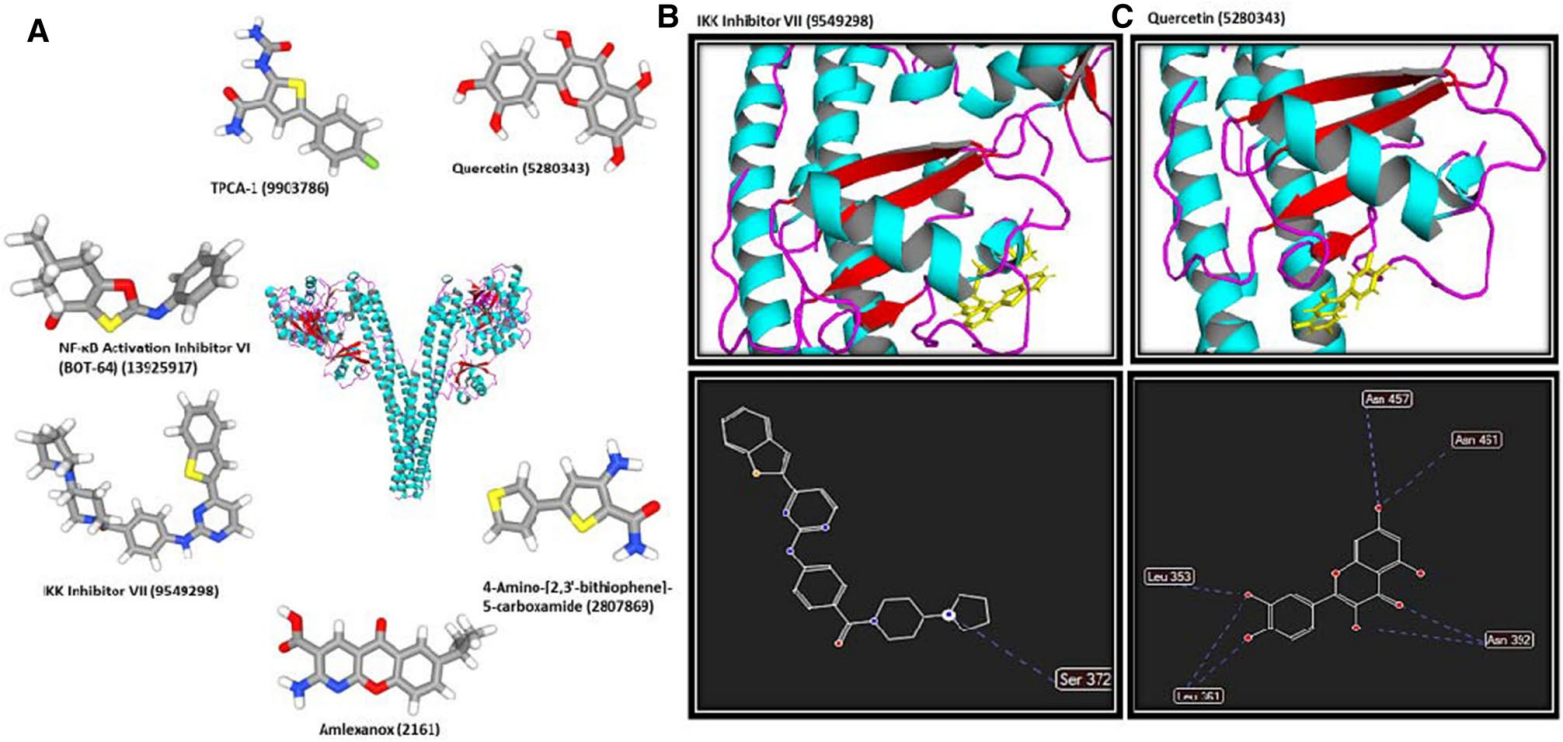
General view of QC-IKK and Inhibitor VII-IKK complexes. (**A**) The structure of all specific inhibitors of IKK are represented. (**B**) Interactions of Inhibitor VII-IKK complex include one hydrogen bond. (**C)** Interactions of the QC-IKK complex contain seven hydrogen bonds.

**Figure 5:**
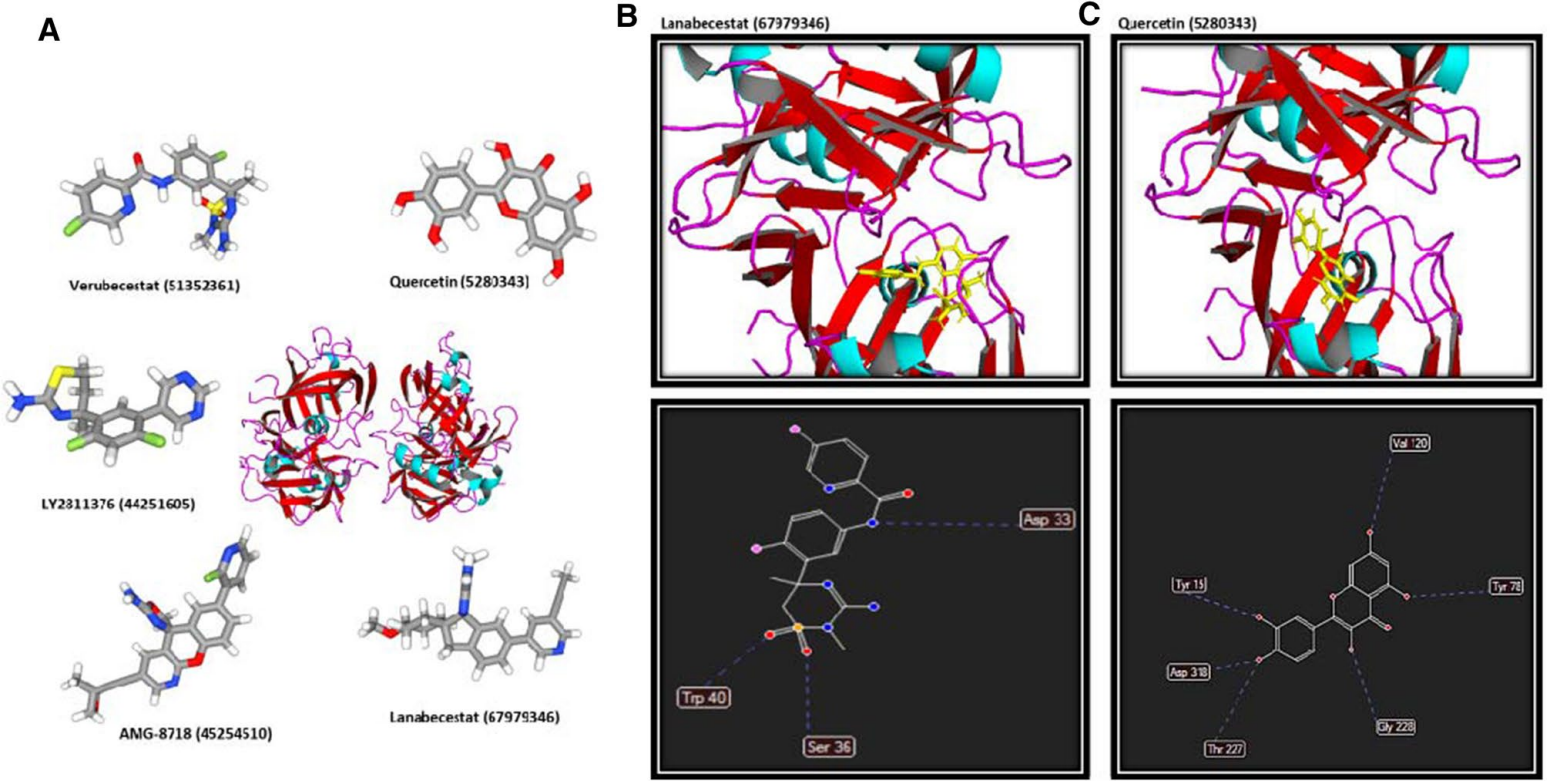
Schematic representation of QC-BACE1 and Lanabecestat-BACE1 complexes. (**A**) The structure of all specific inhibitors of BACE1 are represented. (**B**) Interactions of the Lanabecestat-IKK complex include three hydrogen bonds. (**C**) Interactions of the QC-BACE1 complex contain six hydrogen bonds.

First, five members of the NF-κB pathway including IKK, NF-κB, BACE1, TNF-α, and TRAF6 were selected for further docking analyses. Autodock and Molegro were used to calculate free energy between the proteins, QC, and different previously introduced inhibitors of each protein as distinct ligands. The free binding energies of QC and specific inhibitor of the proteins were calculated (Table 1). A comparison of binding energies of QC and different inhibitors would be helpful to determine if QC plays inhibitory effects on each protein. Docking results obtained from Autodock software for QC and different inhibitors of each protein indicate that the lowest binding energy of QC compared to other inhibitors was obtained by interacting with IKK and BACE1 as − 9.046 and − 9.34 kcal/mol respectively. The consistent results were also obtained from Molegro software for IKK-QC and BACE1-QC as − 87.3986 and − 98.5423 kcal/mol respectively compared to other inhibitors of IKK and BACE1. The structure of all studied inhibitors of IKK are represented in Fig. 4A. Schematic representations of interactions of Inhibitor VII-IKK (Fig. 4B) and QC-IKK (Fig. 4C) complexes where represented. Interactions of Inhibitor VII-IKK and QC-IKK complexes include one and seven hydrogen bonds respectively. Inhibitor VII-IKK complex was selected for representation as it contains the lowest binding free energy compared to other inhibitors of IKK. Furthermore, the structure of all studied inhibitors of BACE1 are represented in Fig. 5A. General view of interactions of Lanabecestat-BACE1 (Fig. 5B) and QC-BACE1 (Fig. 5C) complexes where represented. Interactions of Lanabecestat-BACE1 and QC- BACE1 complexes contain three and six hydrogen bonds respectively. Lanabecestat-BACE1 complex was selected for representation as it contains the lowest binding free energy compared to other inhibitors of BACE1. Therefore, QC would be suggested as a better inhibitor for IKK and BACE1, as the interaction energy between QC and both proteins was lower than the binding energy between the protein and different inhibitors targeted IKK and BACE1. However, results obtained for other 3 proteins including NF-κB, TNF-α, and TRAF6 indicate that the binding energy between proteins and its specific inhibitors was lower than or similar to protein-QC. So, in these cases, QC is not proposed as an esurient inhibitor. According to our results, it can be proposed that the effects of QC on the learning and memory improvement could partly happen through inhibition of NF-кB pathway proteins.

**Table 1:** The docking results based on the binding free energy of QC and specific drugs docked into the involved proteins of the NF-κB signaling pathway using Autodock and Molegro software.

		KEGG entry	PDB ID	QC (PubChem ID 5,280,343)		PubChem ID of Inhibitors	Inhibitors	
				Molegro (MolDock Score (Kcal/mol))	Autodock (kcal/mol)		Molegro (MolDock Score (Kcal/mol))	Autodock (kcal/mol)
1	IKK	K07209	4KIK	− 87.3986	− 9.046	TPCA-1 (9,903,786)	− 85.6963	− 8.456
						NF-κB Activation Inhibitor VI (BOT-64) (13925917)	− 77.3693	− 7.675
						Amlexanox (2161)	− 76.9988	− 8.234
						4-Amino-[2,3′-bithiophene]-5-carboxamide (2807869)	− 84.234	− 7.564
						IKK Inhibitor VII (9549298)	− 86.3455	− 8.487
2	NF-κB	K02580	1NFK	− 98.4567	− 8.982	BAY 11-7082 (5353432)	− 83.4563	− 8.102
						QNZ (EVP4593) (509554)	− 109.3254	− 9.340
						LY294002 (3973)	− 92.4356	− 8.457
						Wortmannin (312145)	− 110.4356	− 9.124
						Mesalamine (4075)	− 106.9831	− 9.342
3	BACE1	K 23621	5UX4	− 98.5423	− 9.34	Verubecestat (51352361)	− 94.5234	− 8.78
						AMG-8718 (45254510)	− 91.7643	− 9.123
						LY2811376 (44251605)	− 76.4532	− 7.453
						Lanabecestat (67979346)	− 95.7643	− 9.135
4	TNF-α	K7124	2AZ5	− 99.5432	− 9.424	Etanercept (7847807)	− 120.4532	− 11.234
						Infliximab (17396768)	− 110.3464	− 10.453
						Golimumab (135329319)	− 98.4356	− 9.456
						Certolizumab (135347437)	− 97.5463	− 8.698
						Adalimumab (135335742)	− 95.3421	− 8.432
5	TRAF6	K03175	4z8m	− 96.4532	− 8.3424	AC1LL9Z5 (1061998)	− 98.3425	− 9.0982
						C25-140 (386330971)	− 100.3453	− 9.431
						CD40-TRAF6 signaling inhibitor (91714451)	− 101.9824	− 10.425

## Discussion

There are several valid reports indicating DM intensifies the risk of cognitive dysfunction and dementia^43, 44^. Dietary flavonoids due to their biological properties may provide supplementary treatments for various complications of DM^45^. Our previous study revealed diabetes impaired spatial learning and memory in the passive avoidance task and Morris water maze. Moreover, QCSPIONs improved learning and memory in diabetic rats more efficiently than pure QC and minimal toxicity on body tissues^38^. To verify and complete the information obtained from this study and propose potential combined therapy to improving learning and memory, the goal of the current research was to clarify a potential molecular change involved in the observed results. First, it should be considered whether these SPIONs cross the BBB or not. Therefore, the quantitative and qualitative contents of the nanoparticles in the nervous system of diabetic rats were calculated with ICP-AES analysis and Prussian blue staining, respectively. Both ICP-AES and Prussian blue staining results demonstrated that SPIONs can cross the BBB and internalize in the nervous system cells (Fig. 1). Cells may be uptake NPs by phagocytosis, various types of diffusion, or endocytosis^46^. In neurodegenerative diseases, the BBB may damage and this may facilitate the entrance of SPIONs to the brain endothelial cells of rats^47^. According to these outcomes and the relationship between DM and BBB dysfunction^48^, it can be concluded that diabetes, through damage to the brain endothelial cells, facilitates the entry of nanoparticles into brain cells.

**Figure 6:**
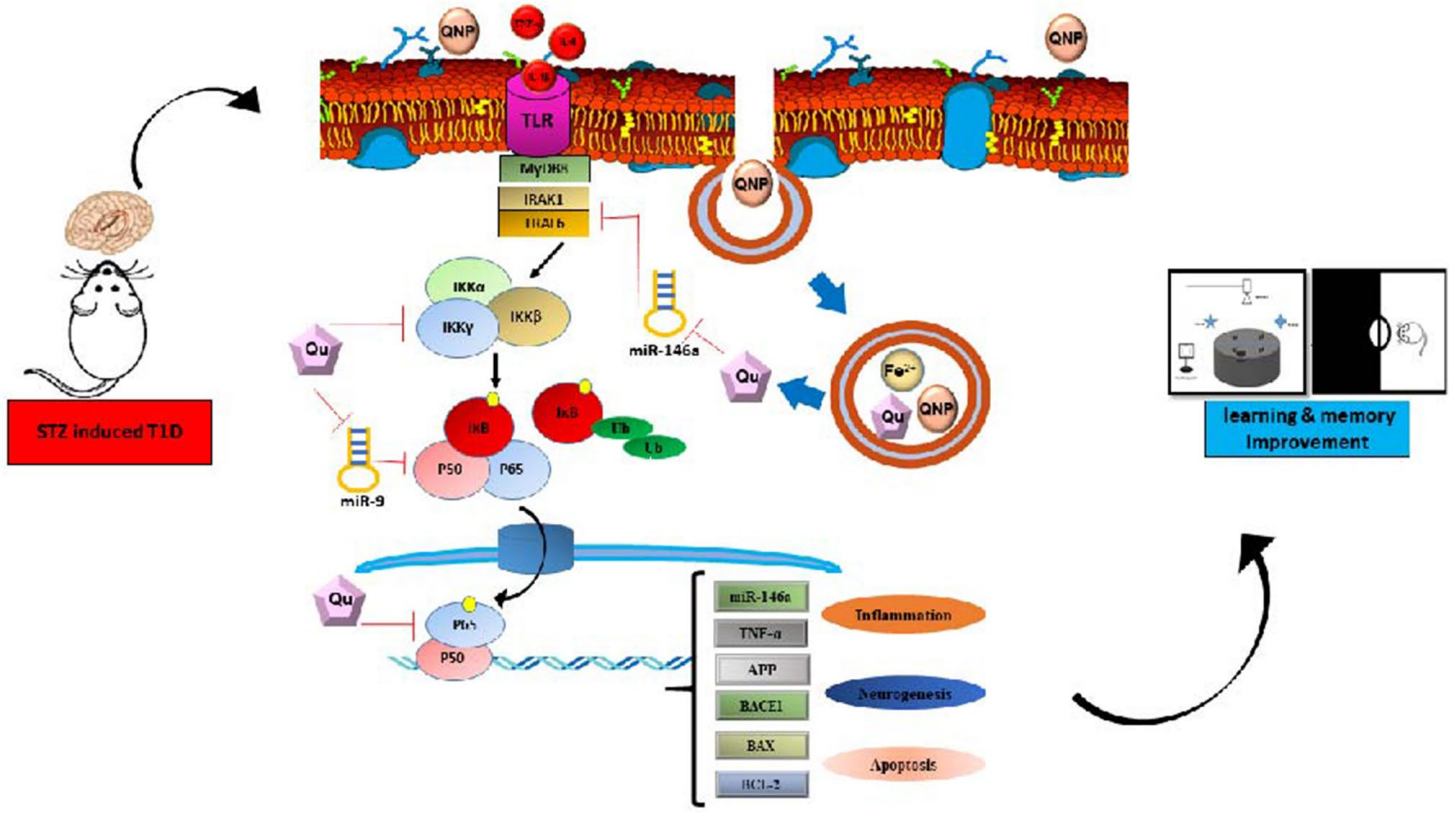
Schematic picture of the beneficial effect of QC after released from SPIONs in neural cells on the miRNAs/NF-κB-dependent inflammatory pathway in the hippocampus of diabetic rats. STZ, streptozotocin; TLR, Toll-like receptors; MYD88, Myeloid differentiation primary response gene 88; IRAK, Interleukin 1 Receptor Associated Kinase 1; TRAF6, TNF receptor-associated factor 6; IKK, Inhibitor of nuclear factor Kappa-B kinase; NF-κB, Nuclear factor-kappa BAPP, Amyloid precursor protein; BACE1, β-site amyloid precursor protein cleaving enzyme 1; BCL-2, B-cell lymphoma 2; BAX, BCL2-associated X protein.

In the present study, we suggested a miRNAs/NF-кB-dependent anti-inflammatory mechanism for justifying the neuroprotective effects of QC in pure and conjugated forms in learning and memory (Fig. 6). TNF-α is one of the most important pro-inflammatory cytokines that can be induced by hyperglycemia and increase the transcription factor NF-κB via stimulation of TNF-α receptors on the surface of the neurons and glia cells^49^. NF-κB has various functions in the CNS, depending on the cell-type. NF-κB is an important regulator of memory, although its direct response to memory enhancement or deficit is inconsistent^50^. In neurons, NF-κB implicates in synaptic plasticity, neuroprotection, neuronal transmission, and it plays a crucial role in converting short-term to long-term memory^51^. Kaltschmidt and colleague revealed that inhibition of NF-κB by repressor IkB or Knockout of p65 in neuronal cells led to defects in neuroprotection and loss of learning and memory. In glia, inducible NF-κB regulates inflammatory processes and aggravates diseases including ischemia, and Alzheimer’s disease (AD). Collectively, neuronal activation of NF-κB can increase memory, whereas glia inhibition of NF-κB might ameliorate disease^52^. In pathological conditions such as DM, impairment to the NF-κB signaling triggers neuroinflammation, microglial activation, oxidative stress, and cell death. These imbalances result in brain homeostatic abnormalities, neuronal degeneration that essentially trigger AD initial stages^53^. The results obtained from the present study revealed that diabetes significantly upregulated mRNA expression levels of TNF-α, NF-кB, and the activity of NF-кB. These data are in line with previous studies, which have reported that NF-κB increased in the hippocampus of diabetic rats^19, 54, 55^. Expression analysis showed that QC and QCSPIONs approximately had a similarly significant effect on the reduction of both TNF-α and NF-кB at the mRNA level; however, QCSPIONs were able to normalize NF-кB activity better than QC (Figs. 2 and 3). These findings are in agreement with Jung et al. who revealed administration of QC reduced neuroinflammation in an animal model of AD by reducing TNF-α, IL-6, IL-1β, and MCP-1^56^. Lu et al. in the same year observed oral administration of QC remarkably ameliorated the behavioral parameters of high-cholesterol-fed old mice through preventing the translocation of NF-кB into nucleus^57^. As shown in photomicrographs of immunohistochemistry staining with antiphospho-NF-κB p65 antibody (Fig. 3), there is a significant reduction in NF-κB activity after treatments, but this is not a complete inhibition that indicates the fundamental role of NF-κB in brain physiology. The constitutive activity of NF-кB initially identified by Kaltschmidt and colleague within neurons from the hippocampus and the cerebral cortex using electrophoretic mobility shift assay (EMSA) and immunofluorescence^58^. In 2000 Albensi and colleague suggested important roles for the involvement of TNF-α and NF-κB activity in the regulation of hippocampal synaptic plasticity^59^. In 2002 Butler et al. used immunostaining and EMSA to measure the activated forms of NF-kB in rat hippocampal slices and showed the physiological and pathological roles of NF-kB in the brain^60^.

Activation of NF‐κβ is also responsible for increasing the level of several types of miRNA such as miR-155, miR-125b, miR-9, and miRNA 146a in the brain^53^. A growing body of evidence showed NFκB activity was regulated through a negative feedback loop in which, NFκB activation induced miR-146a that upon processing and maturation down-regulates two main adapter molecules including TRAF6 and IRAK1 in the TLR/NF-κB pathway to decrease the activity of NF-κB^18, 19, 21^. In this study, miR-146a expression level in diabetic rats was tripled compared to the control group parallel to the overexpression of NF-кB (Fig. 2), suggesting that brain cells were confronting with pathological inflammation and attempting to restore homeostasis. Administration of QC in pure and conjugated forms significantly decreased miR-146a expression level, suggesting the anti-inflammatory effect of QC by miRNA regulation. From the literature review, we found two reports that considered miR-146a as a molecular target for QC; a report from Noratto et al. which indicated a potential role of QC in the upregulation of miR-146a and induction of apoptosis in colon cancer cells^61^ and another report from Tao et al. in 2015 which demonstrated QC prevented proliferation and triggered apoptosis in human breast cancer cells through upregulating miR-146a expression^62^. According to these results, it could be suggested that QC exhibited a bilateral role against miR-146a expression level. When miR-146a is downregulated in different types of cancer, QC can arrest cell proliferation and trigger apoptosis via the upregulation of miR-146a expression, while in diabetes, QC successfully reduces excessive expression of miR-146a to modulate pathological inflammation. Additionally, according to Wang's findings (2016), which indicated dysregulation of miR-146a contributed in tau hyperphosphorylation and AD pathogenesis^63^, we suggest that inhibition of this microRNA by QC treatment could be an in vivo novel therapy for cognitive disorders. Furthermore, our results showed a significant upregulation of miR-9 in the hippocampus of diabetic rats (Fig. 2) which is another response to the excessive expression of NF-κB and the abnormal inflammatory condition, because miR-9 can directly regulate NF-κB. Excessive expression of miR-9 may also be responsible for overexpression of BACE1 because modulation of BACE1 by miR-9 has been previously reported as the role of this miRNA in AD development^64^. Interestingly, the QCSPIONs-treated group showed a natural expression level of miR-9 (Fig. 2), which could be a reason for the higher effect of QCSPIONs on the learning and memory of diabetic rats.

A number of studies have revealed that NF-κB can play a role in processing AβPP and Aβ production via induction the AβPP and BACE1 gene expression^65, 66^. AβPP and BACE1 are recognized as neuronal gene targets of NF-κB. In normal physiology, NF-κB appears to regulate both APP and Aβ negatively. Under pathological conditions, NF-κB activation upregulates promotor activity of AβPP and BACE1 which results in elevated Aβ levels^67^. Our results indicated that hyperglycemia interestingly induced the upregulation of both AβPP and BACE1 mRNA expression levels (Fig. 2) that confirmed a common link between diabetes and cognitive dysfunction. In this line, previous studies suggested that diabetes or IR can stimulate Aβ accumulation via NF‐κβ induction and AβPP and BACE1 overexpression^68^. QCSPIONs were more effective than pure QC in reducing the expression levels of AβPP and BACE1. This could be another reason for the higher efficacy of QCSPIONs to improving the learning and memory of diabetic rats.

The role of the NF-κB in cell survival and apoptosis is very complex and depends on the type of cell in the CNS^69^. Normal NF-кB activity upon inflammation is generally beneficial and inhibits further cell damage while its overexpression is harmful and induces apoptosis in the cells^19^. Bcl-2 and Bax are two key elements of apoptosis in the brain, as reported, the apoptosis rate has an inverted ratio with the expression level of Bcl-2^70^. Here, we observed that the Bax/Bcl-2 expression ratio in the hippocampus of diabetic rats was significantly increased (3.22/2.10 = 1.52), resulting in cell apoptosis. The induction of apoptosis and loss of neuronal in the hippocampus of animal models and diabetic patients have been reported previously^70–73^. Treatment with QC and QCSPION significantly reduced the mRNA expression levels of Bax and Bcl-2 and normalized the Bax/Bcl-2 expression ratio to the control level (Fig. 2). Since the inhibitory effects of QC on the progression of apoptosis is in agreement with previous reports^74–76^, it can be concluded that inhibition of the neural cell death is another possible mechanism of QC and QCSPION on improving learning and memory.

Furthermore, to evaluate the effects of QC on the NF-κB pathway at protein level, docking calculations were performed for five members of the pathway which were introduced previously for targeting with efficient inhibitors. In our study, IKK, NF-κB, BACE1, TNF-α, and TRAF6 were evaluated through docking calculations. Our results indicate that QC could affect the NF-κB pathway at protein level through targeting IKK and BACE1 efficiently compared to the most effective previously introduced inhibitors. IKK is identified as one of the critical regulators of NF-κB activation which is a target for designing therapeutic small molecules^77, 78^. BACE1 is also introduced as one of the key targets for the treatment of AD^79^. So, QC could improve learning and memory by targeting IKK and BACE1 at protein level and regulation of the NF-кB pathway.

In conclusion, dysregulation of miR-146a/miR-9/NF-κB-dependent inflammatory signaling and followed by enhancement expression levels of the AβPP, BACE1, and the ratio of Bax to Bcl-2 in the hippocampus may be an underlying mechanism in diabetes-related memory impairment. QCSPIONs could improve learning and memory by changing the miR-146a, miR-9, TNF-α, and NF-кB expression levels. Additionally, QCSPIONs could able to decrease the pathological activity of NF-кB which resulted in the normalization of AβPP, BACE1, and Bax expression levels. Despite the limitations of this study performed on an animal model of diabetes, we hope these initial findings provide an in vivo promising evidence for the protective and anti-inflammatory roles of QCSPIONs on improving learning and memory.

## Methods

### Synthesis and characterization of QCSPIONs

In order to prepare dextran-coated Fe_3_O_4_ nanoparticles, a chemical co-precipitation method was used, as described in our previous study^38^. Briefly, anhydrous FeCl_3_, FeCl_2,_ and dextran were dissolved in deionized water (DI). After complete mixing, the pH of the mixture was adjusted on 9 using ammonia solution. The mixture was heated at 90ºC for 2 h in consort with stirring, and then through a strong external magnet, the resultant dextran-coated Fe_3_O_4_ nanoparticles were collected. After washing with deionized water and ethanol, the nanoparticles dehydrated in an oven at 70ºC overnight. QCSPIONs were prepared by adding QC to dextran-coated Fe_3_O_4_ nanoparticles and using EDC/NHS as linkers. Then, synthesized QCSPIONs were sequestered from suspension by a magnet. After washing with deionized water, and acetone and QCSPIONs were dehydrated using freeze drier.

Dextran-coated Fe_3_O_4_ nanoparticles and QCSPIONs were characterized by different analytical techniques including FTIR spectrometry and XRD. To determine the size and shape of the nanoparticles, a Hitachi S–4700 FE–SEM, equipped with an energy dispersive X-ray analysis (EDAX) detector was used.

### Experimental induction of diabetes and treatment schedule

Animal maintenance, diabetes induction, and treatment schedule were explained in the previous study^38^. Briefly, adult male Wistar rats (n = 40), weighing 200–230 g were bought from Royan Institute (Isfahan, Iran). They were kept in the animal holding room with the standard condition (40%–50% humidity, 25 °C ± 2 °C temperature, and a 12-h light/dark cycle) for two months. Water and diet were available for all rats during the experiment. To induce type 1 diabetes, streptozotocin (STZ) was injected intraperitoneally for 5 consecutive days at a dose of 20 mg kg^−1^. Animals randomly divided into five groups, including eight rats each: diabetes, diabetes gavaged with SPIONs, QC, or QCSPIONs, and control. All formulations (dose of 25 mg kg ^−1^) were suspended in DI water immediately before administration and gavaged at a daily dose for a period of 35 consecutive days. At the end of the experiment, rats were sacrificed by simultaneous injection a mixture of ketamine (100 mg kg ^−1^) and xylazine (10 mg kg ^−1^). The hippocampus was removed from the hemispheres and ½ of that immediately frozen in liquid nitrogen and then stored at − 70 °C until use. The other half of the hippocampus and other tissues were fixed in 10% formalin and routine paraffin sections (3–4 μm) were preserved for histopathologic evaluation. The ethical respects were done according to the guidelines for the use and care of research laboratory animals (USA National Institute of Health Publication No 80-23, revised 1996) and all experimental protocols were approved by the animal ethics committee of the University of Isfahan.

### The entrance of SPIONs into tissues

The qualitative distribution of nanoparticles in the tissue sections of rats was performed by Prussian blue staining. The tissues, including pancreases, liver, kidney, and hippocampus were removed and soaked in 10% formalin for at least 24 h. Sections with 4 μm thickness were prepared and stained with Prussian blue according to the standard laboratory protocols. Briefly, the sections were hydrated and then dipped in a 1:1 acid solution of 10% potassium ferrocyanide (K4Fe[CN]6) and 20% hydrochloric acid (HCl) for 15 min. In this reaction, ferric ion (+ 3) in the tissue is released by HCl and in combination with the ferrocyanide produces a bright blue pigment termed ferric ferrocyanide or ''Prussian blue" that gives suitable contrast in the optical microscopes. Sections were rinsed with ddH_2_O and counterstained with nuclear fast red. The sections were dehydrated and mounted with DPX medium, coverslipped. Finally, a light microscope (Inverted Microscope, Nikon, Tokyo, Japan) was used to examine images of stained sections.

In addition to Prussian blue staining, ICP-AES) (ICPS-7500, Shimadzu, Japan) was performed to determine the quantitative concentration of SPIONs in the brain as the target tissue in this study. To achieve this purpose, an equal amount of samples was individually exposed to 3 mL nitric acid (HNO3, 65%) overnight. After digestion, resultant mixtures were filtered and iron concentration analyzed using ICP- AES technique and reported as a graph.

### RNA extraction and complementary DNA (cDNA) synthesis

In order to extract RNA, 50–100 mg of tissue was taken from each animal and squashed in a sterile Petri. Regarding the manufacturer’s procedure, TRIzol Reagent (Invitrogen, Life Technologies, Grand Island, NY, USA) was used to extract total cellular RNA (including messenger RNA and microRNA). The concentration, purity, and integrity of the RNA samples were assessed by a Nanodrop spectrophotometer (Thermo Fisher Scientific, USA) and denaturing agarose gel electrophoresis. DNA contamination was removed from RNA samples by treating 1 μg of the RNA samples with 1 unite RNAase-free DNase (Thermo Fisher Scientific Inc, USA).

To synthesize cDNA from total RNA, a PrimeScript RT reagent kit (Takara Bio, Ohtsu, Japan) was used in a final volume of 10 μL. 2 μL 5 × PrimeScript buffer, 0.5 μL oligo dT primer, 0.5 μL RT enzyme, 0.5 μL of random 6mer and 500 ng DNase- treated total RNA were mixed and incubated for 15 min at 37 °C and 5 s at 85 °C, respectively. In order to prepare cDNA from microRNAs, a BON-miR miRNA 1st strand cDNA synthesis kit (Bonyakhteh, Tehran, Iran) was applied. Then, elongations of miRNAs were briefly performed in a polyadenylation reaction with a final volume of 20 μL at 37 °C for 30 min. To stop the reaction, miRNAs were incubated at 65 °C for 20 min. Then, the cDNA synthesis reaction was accomplished in a final volume of 20 μL by adding 1 μL RT enzyme, 1 μM Bon-RT adaptor, 4 μL 5 × RT buffer, 10 μL polyadenylated total RNA, and 2 μL dNTP mix. Finally, reactions were incubated at 75, 25, 42, and 70 °C, for 5, 10, 60, and 10 min, respectively.

### Quantitative real-time PCR

Each synthesized cDNA was applied as a template for a distinct microRNA and mRNA quantitative real-time PCR assay. β-actin and U78 were chosen as internal housekeeping for normalization mRNA and microRNA expression, respectively. All primers for mRNA samples were designed using Allele ID primer design software version 7.5 (Premier Biosoft, USA) (Table 2). Then investigated in the NCBI website (www.ncbi.nlm.nih.gov/blast) and purchased from BIONEER (City, Korea). Locked nucleic acid (LNA) forward and universal reverse primer for microRNA samples were set by Bonyakhteh Company (Bonyakhteh, Tehran, Iran). The real-time PCR assay for mRNAs was carried out in a final reaction volume of 10 μL containing 5 μL RealQ Plus 2 × Master Mix Green (Ampliqon, Odense, Denmark), 0.5 μM of 10 pM forward, 0.5 μM of 10 pM reverse primers and 1 μl cDNA containing 10 ng cDNA. The assay was done on a Bio-Rad Detection System (Bio-Rad, USA) using the following cycling conditions: 15 s at 95 °C as the first denaturation step, followed by 39 cycles at 95 °C for 15 s and 57 °C for 30 s and 72 °C for 15 s. Quantitative real-time PCR for miRNAs analysis was performed using BON microRNA quantitative PCR Master mix kit in a final reaction volume of 13 μL containing 0.5 μL universal reverse primer,0.5 μL miRNA specific forward primer, 6.5 μL quantitative PCR master mix, and 1 μL cDNA. To assay real-time PCRs the following cycling conditions were chosen: 95 °C for 2 min, followed by 40 cycles of 95 °C for 5 s and 60 °C for 30 s. Relative differences in gene expression levels were calculated by the 2^−ΔΔCt^ method. To evaluate the efficiency of primers, standard curves were designed by plotting the threshold value (CT) against the log of the amount of total cDNA used in each reaction.

**Table 2:** Primers for Real-time PCR.

Target bp^a^	Forward primer 5′ → 3'	Reverse primer 5′ → 3'	Amplicon bp^a^
β-actin	CTCTATGCCAACACAGTG	AGGAGGAGCAATGATCTT	123
NF-κB	TTACGGGAGATGTGAAGAT	ATGATGGCTAAGTGTAGGA	94
TNF-α	GTGTTCATCCGTTCTCTAC	CCACAATTCCCTTTCTAAGT	123
BACE1	GAAGTCACCAATCAGTCC	ACTTGTAACAGTCGTCTTG	97
AβPP	TACTGCCAAGAGGTCTAC	CGGTAAGGAATCACGATG	134
BAX	TTTGCTACAGGGTTTCATC	ATGTTGTTGTCCAGTTCAT	147
Bcl-2	GTGGATGACTGAGTACCT	GCCAGGAGAAATCAAACA	119

^a^Base pair.

### Immunohistochemistry

Immunohistochemistry was performed to detect the phospho NF-κB p65 in the hippocampus. Deparaffinization and rehydration were done by placing Sects. (3 μm) in containers of xylene and ethanol several times. The slides were incubated in buffer with pH 9 and placed in the microwave for 20 min. The sections incubated in 3% H2O2 at 37 °C for 10 min to quench the endogenous peroxidase activity. Then, the slides were incubated with 5% normal goat serum for 30 min to block the nonspecific epitopes. After washing with Phosphate-buffered saline (PBS), the sections incubated overnight with rabbit polyclonal antibody NF-κB p65 (phospho S536) (NF-κB, ab28856) (diluted 1:100) at 4 °C in a humidified chamber. After washing three times with PBS, the slides were exposed to biotinylated goat anti-mouse/rabbit IgG (diluted1: 100) 1 h in the dark room, followed by streptavidin-peroxidase for 1 h at room temperature. The slides were rinsed and then treated with diaminobenzidine tetrahydrochloride^33^ as the substrate and counterstained with hematoxylin. NF-κB reactivity was detected by the evaluation of five randomly selected areas under a light and a fluorescent microscope equipped with a digital camera (Olympus SZX12 fluorescence stereo zoom microscope, Japan). The positive reaction of NF-κB in each photograph was evaluated and the average of all photographs in each group was calculated.

### Computational analysis

#### Preparation of protein and ligands structure

All proteins of the NF-кB pathway were studied to find out core members of the pathway which are critical targets for drug design. Among all, NF-кB, IKK, BACE1, TNF-α, and TRAF6 were selected for further analyses. For each protein, the most effective drugs were also selected using PubChem, PubMed, ScienceDirect. The molecular structures of all proteins were obtained from RCSB Protein Databank (PDB). In order to get more accurate results, the protein structure was refined prior to docking analysis. The original bound ligands, as well as crystallographic water molecules, were removed and then the structures of proteins were saved in the pdb format. The structures of QC and all specific inhibitors of each protein were obtained from PubChem (https://pubchem.ncbi.nlm.nih.gov/) in sdf format.

#### Docking simulations

The molecular docking simulation was used to find the optimal conformation and orientation of different ligands when they interact with proteins. In this molecular docking study, AutoDock 4.2.6 and Molegro Virtual Docker (MVD) version 6.0 2014 by CLC bio Company along with Graphical User Interface, MVD tools were utilized.

AutoDock is identified as a well-known docking program for calculation of the interactions between proteins and ligands using empirical free energy force field^80^. The AutoDock tools were used to create PDBQC format of ligands and proteins. The AutoGrid was used for grid maps calculation and three-dimensional grids of 64 × 64 × 64 Å3 were set on ligand binding site. The docking parameters were used as follow: Lamarckian genetic algorithm (LGA) consisted of 200 runs, 250,000 energy evaluations, the initial population of 150 randomly generated individuals, a mutation rate of 0.02, a maximum of 27,000 generations and a crossover rate of 0.80. For selecting the best model, the free binding energy and 10 best ligands positions were analyzed.

Furthermore, MVD was used for more validation and visualization of the schematic plan of ligand hydrogen bonds. MVD has been recently gained attention for predicting protein–ligand interactions among medicinal chemists^81^. First, the charge was calculated by MVD and flexible torsions in ligands were detected during the preparation process. Then, potential missing bonds were assigned and possible explicit hydrogens were generated. The side chain minimization was also performed. The Piecewise Linear Potential (PLP) scoring functions were used for MolDock scoring. Default settings were utilized for all calculations. Docking calculation was performed for 10 independent runs using a grid resolution of 0.30 A^o^. Furthermore, PyMOL (Molecular Graphics System, Version 2.3.2, Schrodinger, LLC) and Molegro packages were also utilized in order to visualization and representation of the proteins, chemicals, and protein-chemical complexes.
